# The effect of plant identity and mixed feeding on the detection of seed DNA in regurgitates of carabid beetles

**DOI:** 10.1002/ece3.4536

**Published:** 2018-10-25

**Authors:** Daniela Sint, Yasemin Guenay, Rebecca Mayer, Michael Traugott, Corinna Wallinger

**Affiliations:** ^1^ Mountain Agriculture Research Unit, Institute of Ecology University of Innsbruck Innsbruck Austria; ^2^ Institute of Interdisciplinary Mountain Research, IGF Austrian Academy of Sciences Innsbruck Austria

**Keywords:** Carabidae, feeding experiment, granivory, *Harpalus rufipes*, omnivory, seed predation, *trn*L

## Abstract

Carabids are abundant in temperate agroecosystems and play a pivotal role as biocontrol agents for weed seed and pest regulation. While there is good knowledge regarding their effects on invertebrate pests, direct evidence for seed predation in the field is missing. Molecular approaches are ideally suited to investigate these feeding interactions; however, the effects of an omnivorous diet, which is characteristic for many carabid species, and seed identity on the detection success of seed DNA has not yet been investigated. In a series of feeding experiments, seeds of six different Central European weed species were fed to beetles of the species *Pseudoophonus rufipes*, to determine post‐feeding seed DNA detection rates and how these are affected by plant identity, meal size, and chemical seed composition. Moreover, we investigated the effect of a mixed diet of seeds and mealworm on prey DNA detection. Four out of six seed species were detectable for up to five days after consumption, and seed species identity significantly affected post‐feeding detection rates. Detectability was negatively influenced by protein content and seed mass, whereas oil content and meal size had a positive effect. The mixed diet led to both increased detection rates and post‐feeding detection intervals of seed DNA. This suggests that mixed feeding leads to an enhancement of food detection intervals in carabid beetles and that seed identity, their chemical composition, and meal size can affect DNA detection of consumed seeds. These aspects and potential implications of this non‐invasive approach are discussed as they can become highly relevant for interpreting field‐derived data.

## 1 INTRODUCTION

During the last decades, there has been an increasing demand for food and accordingly agricultural productivity. Although agricultural intensification—including reduction of habitat complexity, intense fertilization, and pesticide application—has substantially increased crop yield, they are also coupled with higher production costs, pesticide resistance, and negative impacts on ecosystems and human health (Matson, Parton, Power, & Swift, 1997; Tilman, Cassman, Matson, Naylor, & Polasky, 2002). To reduce such negative effects and to make farming sustainable, it is important to implement strategies which boost the efficacy of ecosystem services such as the regulation of crop pests and weeds by natural enemies (Bommarco, Kleijn, & Potts, 2013; Naylor & Ehrlich, 1997). Generalist natural enemies such as carabid beetles have been shown that they can be effective biological control agents (Östman, 2004; Symondson, Sunderland, & Greenstone, 2002). Compared to specialists, they are already present in a field at the pest's arrival and thus have the potential to prevent pest outbreaks (Chang & Kareiva, 1999; Symondson, Sunderland, et al., 2002; Wissinger, 1997). Carabids are widely recognized as important beneficial organisms in arable land, known to provide regulation services on aphids (Lang, 2003; Roubinet et al., 2017; Staudacher, Jonsson, & Traugott, 2016), pest slugs (Bohan, Bohan, Glen, Symondson, & Wiltshire, 2000; El‐Danasoury, Cerecedo, Córdoba, & Iglesias‐Piñeiro, 2017; Fusser, Pfister, Entling, & Schirmel, 2016; Symondson, Glen, Ives, Langdon, & Wiltshire, 2002; Thomas, Harwood, Glen, & Symondson, 2009), and weed seeds (Bohan, Boursault, Brooks, & Petit, 2011; Honek, Martinkova, & Jarosik, 2003; Tooley & Brust, 2002). According to a national‐scale study, the turnover of the weed seedbank in individual fields is negatively correlated to the abundance of carabids (Bohan et al., 2011). This indicates their potential impact on the demography of weed species via seed predation (Westerman, Wes, Kropff, & Werf, 2003).

Among omnivorous carabids, *Pseudoophonus* (*Harpalus*)* rufipes* (De Geer, 1774) is a particularly well‐studied species (Harrison & Gallandt, 2012). It is widespread throughout arable systems (Brandmayer, 1990) and usually occupying a dominant position in carabid communities in a wide range of agroecosystems in different countries (Jørgensen & Toft, 1997; Kromp, 1999; Langmaack, Land, & Büchs, 2001; Luff, 2002). *Pseudoophonus rufipes* consumes a broad variety of prey (Jørgensen & Toft, 1997; Kielty, Allen‐Williams, & Underwood, 1999; Kromp, 1999; Monzo, Sabater‐Muñoz, Urbaneja, & Castañera, 2011; Renkema, Manning, & Cutler, 2013), and a large proportion of its diet is made up of seeds (Brandmayer, 1990; Holland, 2002; Larochelle, 1990; Tooley & Brust, 2002). However, carabid feeding behavior is known to be dynamic in response to various physiological and environmental conditions, making it difficult to generalize (Lundgren, Saska, & Honěk, 2013). Despite the comprehensive knowledge on the role of carabids as biocontrol agents in general and the wide potential prey spectrum in the specific case of *P. rufipes*, field data about its actual food choice are scarce. Most of the dietary information stems from laboratory experiments, demonstrating that specific granivorous carabids select for a particular seed species when provided with a limited choice (Honek et al., 2003; Honek, Saska, & Martinkova, 2006). However, such experimental setups might distort their natural feeding behavior (Boursault, 2013; Lundgren, 2009), and the trophic interactions between carabids and specific prey species seem to be highly dynamic and dependent upon environmental conditions (Roubinet et al., 2017).

DNA‐based diet analysis has become a widely used approach for the identification of trophic interactions under natural conditions (Pompanon et al., 2012; Symondson & Harwood, 2014; Traugott, Kamenova, Ruess, Seeber, & Plantegenest, 2013). However, apart from a few exceptions (Lundgren et al., 2013; Lundgren, Ellsbury, & Prischmann, 2009; Wallinger et al., 2015), granivorous carabids have received little attention in this field. The consumption, digestion, and thus detectability of seeds might be essentially different compared to other plant tissue or animal prey. Earlier experiments in this field showed promising results, where seed DNA had been traced in the regurgitates of carabids for at least 3 days post‐feeding (Wallinger et al., 2015), an even longer detection period than previously thought. Among the variety of molecular approaches, next‐generation sequencing (NGS) enables many consumed species to be detected simultaneously via DNA metabarcoding. To date, numerous NGS studies of herbivory have employed general plant primers targeting the *trn*L‐region (Pompanon et al., 2012; Soininen et al., 2009, 2013 ; Valentini, Miquel, et al., 2009; Valentini, Pompanon, & Taberlet, 2009). Continual improvement, ongoing decreases in costs, and current expansion of reference databases make species identification based on the *trn*L‐region a promising approach, also in respect to the detection of trophic links between carabids and specific seed species in arable fields. The present feeding experiments were initiated in view of such field investigations, as differences in the detectability of varying seed species may occur if targeted with general plant primers (Wallinger et al., 2015). So far, we have a poor understanding of how seed characteristics (i.e., species identity, chemical composition) or the meal size affect post‐feeding seed DNA detection intervals via general plant primers in carabid beetles. As such, we have employed general plant primers targeting the *trn*L region for investigating the detectability of different consumed seed species.

Given that the vast majority of carabids is omnivorous and consumes a wide range of food (Lövei & Sunderland, 1996), they may eat both seeds and insect prey at the same time. Dietary mixing of different animal prey showed contradictory influences on the molecular detectability of food in arthropods (Lövei, Sopp, & Sunderland, 1987, 1990 ; Weber & Lundgren, 2009). However, currently there is still no information available on how the DNA detectability of a specific seed is influenced by a mixed diet in carabids, although this information would be highly relevant for the interpretation of field derived molecular trophic data of omnivorous arthropods.

The aims of the present study were to identify (a) the maximum detection time during which seed consumption can be traced molecularly in carabid regurgitates by the example of *P. rufipes*, (b) the impact of seed species identity, seed chemical composition, and meal size on post‐feeding plant DNA detection, and (c) the influence of a mixed diet on the detectability of both consumed seeds and mealworm. To investigate these issues, we established a set of feeding experiments, offering seeds of six different weed species, and a mixed diet to carabids and compared the seed DNA detection success in regurgitates with general plant primers targeting the *trn*L exon (chloroplast DNA), and species‐specific primers targeting the COI gene (mitochondrial DNA) for the animal prey.

## 2 MATERIAL AND METHODS

### 2.1 Species collection and experimental setup

Adult beetles of *P. rufipes* were trapped on arable land near Innsbruck (760 m a.s.l.; Tyrol, Austria) in two successive summers (August 2014 and 2015). The beetles were kept individually in a climate cabinet (L:D 14:10 hr at 22 and 12°C, respectively) in plastic beakers (71 × 58 mm, h × Ø, screw top lid), containing a piece of moistened tissue. Beakers were ventilated and the tissues replaced every second day. The beetles were maintained on a diet of 1/3 piece of mealworms (*Tenebrio molitor L*.), which was offered every fifth day. Prior to the feeding experiments, beetles were starved for five days.

In many plant species, what is regularly referred to as the “seed” is actually an achene, a fruit containing a single seed which does not open at maturity, as it is for *Taraxacum officinale* and *Senecio vulgaris*. In the case of *Lolium perenne,* the grain resembles an achene, except that it is a caryopsis with a seed coat fused to the seed wall. For reasons of simplicity, we will refer to both the achene and the caryopsis as seeds in the text hereafter. For the experiments to test the impact of seed identity, seeds of six plant species were chosen as experimental food: *Capsella bursa‐pastoris, Lolium perenne, Rumex obtusifolius, Senecio vulgaris*,* Taraxacum officinale,* and *Trifolium repens*. These plant species are common weeds in Central European arable land and are known to be eaten by *P. rufipes* (Lundgren, 2009). The seeds of these plants vary significantly in their mass and nutrient composition. For example, the mass of the heaviest seeds (*L. perenne*) was 22 times higher than of the lightest seed (*C. bursa‐pastoris*) and the oil content ranged from 1.8% (*L. perenne*) to 32.7% (*S. vulgaris,* Table 1).

**Table 1:** Seed traits and consumption of the six seed species offered to *Pseudoophonus rufipes* in the species‐identity feeding experiments. Data source for seed traits: Seed Information Data Base SID (https://data.kew.org/sid). As this database does not provide information on *Senecio vulgaris* seeds, information on the oil content stems from Bretagnolle, Matejicek, Gregoire, Reboud, and Gaba (2015); for the estimation of the protein content, we took the mean between *S. ambrosioides* and *S. hieracifolius,* that are comparable in seed size and oil content. Mean seed number represents the average number of seeds consumed by *P. rufipes* during the feeding experiment and the corresponding mean meal size was calculated as mean 1,000 seed mass × mean seed number

Plant species	Mean 1,000 seed mass (g)	Oil content (%)	Protein content (%)	Mean seed number	Mean meal size (mg)
*Capsella bursa‐pastoris*	0.1	30.5	28.2	1.74	0.17
*Lolium perenne*	2.2	1.8	18.8	2.26	4.97
*Rumex obtusifolius*	1.5	2.8	12.7	1.85	2.77
*Senecio vulgaris*	0.2	32.7	20.2	1.80	0.36
*Taraxacum officinale*	0.6	26.7	30.0	2.50	1.50
*Trifolium repens*	0.7	6.3	35.1	1.42	0.99

For the seed‐identity experiment, fresh feeding containers (60 × 35 mm, h × Ø) were prepared with a drop of water and five seeds of a single plant species each. The beetles were put individually into the feeding tubes and allowed to feed for 2 hr in the dark climate cabinet. Afterward, carabids that had consumed at least half a seed were put individually in clean beakers and kept in the climate cabinet until regurgitation without any further food provided. Beetles were stimulated to regurgitate in batches of 10–15 individuals as described in Wallinger et al. (2015) at different time points post‐feeding (0, 16, 32, 64, 96, 128 hr). Detailed information on the number of included individuals per time point for each seed species is provided in Supporting Information Table S1. Regurgitates were immediately frozen and stored at −28°C until DNA extraction. All beetles were set free in their original habitat at the end of the experiments.

For the mixed‐diet experiments, the same general procedure was applied as above. Here, two different seed species were selected as a “starter”: *C. bursa‐pastoris, L. perenne,* and afterward one third of a freshly cut mealworm was offered as a “main course.” As carabid beetles did not eat any seeds after having fed on animal prey as a “starter” (personal observation), we refrained from testing the food sequence the other way round. To avoid potential detection of plant DNA present in the herbivorous *T. molitor* larvae in the carabid regurgitates, mealworms had been starved for 7 days prior freeze killing them for the experiment. Again, beetles were put individually into the feeding tubes and allowed to feed for 2 hr on 5 seeds of either *C. bursa‐pastoris* or *L. perenne* in the dark climate cabinet. Beetles that had consumed at least half of a seed were put individually in fresh beakers and allowed to digest for one (i.e., short between seed and mealworm consumption) and eight hours (long), respectively. Thereafter, they were supplied with one third of a *T. molitor* larva, which was removed after another hour and beetles were put in clean beakers in the climate cabinet until regurgitation. Beetles were stimulated to regurgitate in batches of 10–14 individuals at different time points (0–128 hr) post‐feeding of the mealworm (Supporting Information Table S2).

### 2.2 DNA extraction, PCR, and electrophoresis

For lysis, regurgitates were mixed with 200 µl 1×TES buffer 5 µl Proteinase K (20 mg/ml) and approx. 1 mg PVP (Polyvinylpyrrolidone) and incubated for 3 hr at 58°C. DNA was thereafter extracted with the BioSprint 96 DNA Blood Kit (Qiagen, Hilden, Germany) on a Biosprint^®^ 96 extraction robotic platform (Qiagen) following the manufacturer's instruction, with the exception of the lysis step described above and DNA elution in 200 µl 1× TE buffer. DNA extracts were stored at −28°C. All extractions were conducted in a separate pre‐PCR laboratory using a UVC‐equipped laminar flow hood, and two extraction‐negative controls (PCR‐grade water instead of seeds or regurgitate) were included in each batch of 48 samples to check for cross‐sample contamination during the extraction process. None was detected by testing the controls using the diagnostic assays described below.

To test the regurgitates from the seed‐identity experiments for the presence of plant DNA, all samples were screened with the general plant primers “*g* A49425” and “*d* B49863” (Taberlet et al., 2007). They amplify a 300‐bp fragment from the *trn*L exon, a recommended size for dietary studies (Greenstone, Payton, Weber, & Simmons, 2014). The PCR assay and conditions followed the description provided in Wallinger et al. (2013).

For the mixed‐diet experiments, DNA extracts were tested twice: once for seed DNA with the same primer combination and PCR conditions as in the seed‐identity experiment (described above) and once with the mealworm‐specific primers Ten‐mol‐S210 and Ten‐mol‐A212, amplifying a 128‐bp fragment (Oehm, Juen, Nagiller, Neuhauser, & Traugott, 2011). For the mealworm detection, the 15 µl reactions included 4 µl DNA extract, 7.5 µl 2× Type‐it MutationTM Detect PCR Kit (Qiagen, Hilden, Germany), 0.5 µl 5× Q‐Solution (Qiagen), 0.5 µg bovine serum albumin (BSA), 4 mM MgCl2, and 0.75 µl of each primer. The cycling protocol included 5 min at 95°C, 35 cycles of 20 s at 92°C, 30 s at 60°C, 30 s at 70°C, and a final elongation of 5 min at 70°C. Within each PCR, one negative control (molecular grade water instead of template DNA) and one positive control (seed/mealworm DNA) was run to check for DNA carryover contamination and amplification success, respectively. All PCR products were visualized using QIAxcel, an automated capillary electrophoresis system (Qiagen), with method AL320, and the results were scored with QIAxcel BioCalculator Fast Analysis Software version 3.0 (Qiagen). Samples showing the expected fragment length with a signal strength exceeding 0.1 relative fluorescent units were deemed to be positive. The DNA extracts of the regurgitates that tested negative in a first run were re‐tested in a second PCR, running the original DNA template to increase the chances of amplification in samples containing only minute quantities of food DNA (Sint, Raso, Kaufmann, & Traugott, 2011).

### 2.3 Statistical analyses

An ANOVA with Bonferroni‐corrected post hoc tests was performed to analyze differences in the mean number of consumed seeds between the six seed species. Overall plant DNA detection success was tested for significant differences between seed species, using generalized linear models (GLM, LOGIT link function including digestion time (= time post‐feeding) and the interaction between seed species and time as explanatory factors). Pairwise post hoc comparisons (Wald statistics) of estimated marginal means were performed with digestion time fixed at 0 hr and sequential Bonferroni correction. For the mixed‐diet experiment, the influence of the two seed species, the length of the meal break (long vs. short), digestion time, and the interaction between seed species and time on the detection of food DNA (seed and mealworm DNA, respectively) were analyzed using GLMs. Other interaction terms were removed from the models based on nonsignificant effects and informed by Akaike's information criterion corrected for finite samples (AICC). For comparisons of estimated marginal means, digestion time was fixed at 0 hr.

To analyze how time, seed species‐specific traits, and consumed biomass influence the detection success, a discriminant analysis was performed with “digestion time,” “1,000 seed mass,” “oil content,” “protein content,” and “meal size” (i.e., number of consumed seeds × average seed mass obtained from the 1,000 seed mass), as predictive variables. All calculations were performed in IBM SPSS Statistics 24 (IBM, Armonk, NY, USA).

## 3 RESULTS

In total, 411 regurgitates were tested for seed DNA in the seed‐identity experiment and 307 regurgitates for both seed and mealworm DNA in the mixed‐diet experiment. Overall, the carabid beetles consumed on average 1.97 (±1.00 *SD*) seeds within a 2‐hr feeding period. Between the six plant species, the average number of consumed seeds varied from 1.4 in *T. repens* to 2.5 in *T. officinale,* which resulted in highly significant differences in the meal sizes due to the variation in the 1,000 seed mass (Table 1). Bonferroni‐corrected post hoc tests revealed for all pairwise comparisons highly significant differences (*p* < 0.001), except for *S. vulgaris* versus *T. repens* (*p* = 0.013), *S. vulgaris* versus* C. bursa‐pastoris* (n.s.), and *T. officinale* versus *T. repens* (n.s).

**Figure 1:**
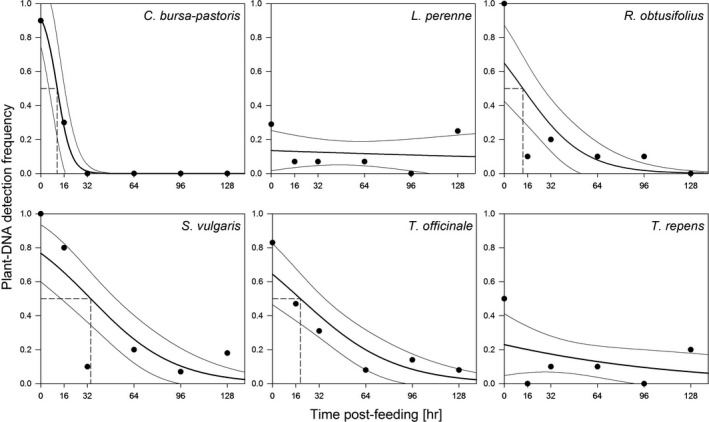
Detectability of seed DNA in regurgitates of *Pseudoophonus rufipes* fed with seeds of six different seed species at different time points post‐feeding ranging from 0 to 128 hr post‐feeding. A minimum of 10 regurgitates per time point post‐feeding was tested with general plant primers. Observed detection rates are provided for the different time points as circles along with fitted LOGIT models for the decrease in seed DNA detection success including the lower and upper 95% confidence intervals (thin lines) and the time points where the detection probability equals 50% (dashed lines)

**Figure 2:**
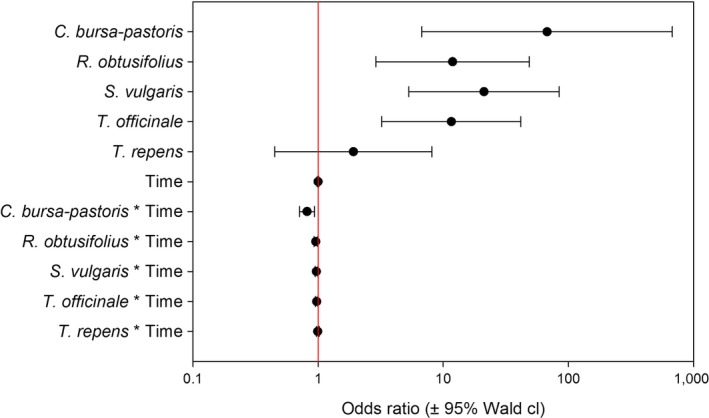
Odds ratios of seed DNA detection compared to *Lolium perenne* (OR = 1; vertical red line) inferred from the LOGIT regression for the different seed species and the interaction between seed species and time

In four out of the six plant species tested, the seed consumption could be detected in the beetle regurgitates for up to 128 hr post‐feeding, the maximum digestion time in our experiments (Figure 1). For *R. obtusifolius,* the maximum detection time was 96 hr post‐feeding, and for *C. bursa‐pastoris,* it was 32 hr only. The detectability of seed DNA in regurgitates decreased significantly with digestion time for *C. bursa‐pastoris, R. obtusifolius, S. vulgaris, T. officinale,* and *T. repens*. No such trend could be observed for *L. perenne* seeds where detection was generally low. The time to reach a predicted detection probability of 50% ranged between 11.3 hr in *C. bursa‐pastoris* and 34.3 hr in *S. vulgaris*. For *L. perenne* and *T. repens* seeds, the model predicted detection rates below 50% already at 0 hr post‐feeding (Figure 1). The comparison of the overall detection rates for the six plant species with the GLM showed significant main effects for both, “seed species identity” (Wald χ^2^ = 32.922, *p* < 0.001) and “time post‐feeding” (Wald χ^2^ = 20.163, *p* < 0.001) as well as a weak but significant interaction of “seed species” and time (Wald χ^2^ = 20.693, *p* = 0.001). A comparison of the odds ratios for seed DNA detection indicates an elevated detection probability for *C. bursa‐pastoris*,* R. obtusifolius, S. vulgaris,* and *T officinale* compared to *L. perenne* (Figure 2)*.* Although also the odds for *T. repens* to detect seed DNA are 1.91 times the ones for *L. perenne*, the wide confidence intervals including one, do not allow inferring a difference between these two seed species. These findings were also supported by the pairwise comparison of the estimated marginal means for the different seed species, where two species cluster appeared. *Lolium perenne* and *T. repens* showed significantly lower predicted detection rates than the other four species (*T. repens* vs. *R. obtusifolius p* = 0.035, *T. repens* vs. *T officinale p* = 0.013, all other pairs between the two clusters *p* ≤ 0.001).

**Figure 3:**
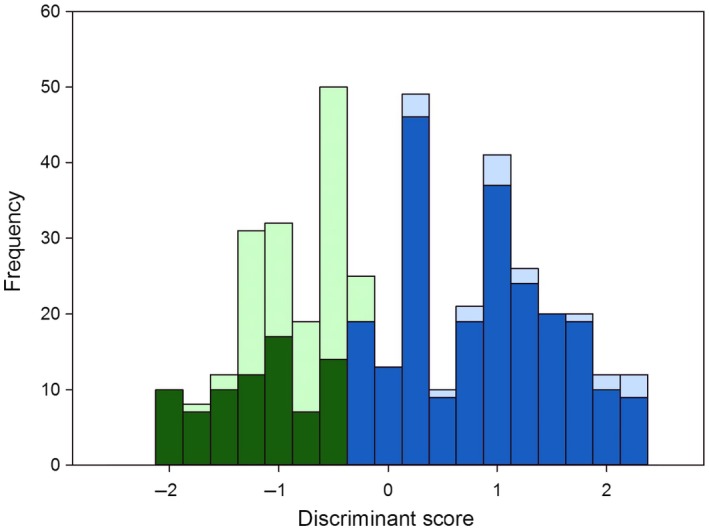
Histogram of the discriminant scores for samples being classified by the discriminant analysis as positive (green; discriminant score below −0.3) or negative (blue; discriminant score above −0.3). Dark colors indicate correct classification. Light colors misclassification compared to the actual screening results

The eigenvalue of the discriminant analysis to investigate the influence of meal size and seed‐specific traits on the detection probability of seed DNA was 0.229, which is rather low. However, based on the analysis, a total of 73.5% of the regurgitates (302 out of 411 samples) were correctly classified as either “plant DNA detectable” or not (Table 2). While 81.1% of the positive detections were correctly classified by the discriminant analysis (77 out of 95), the classification success for negative detections was only 71.2% (225 out of 316). The cross‐validation showed a very small dependency on individual samples, with just two more false‐positive classifications. The group centroids of the calculated discriminant scores were −0.87 for positive and 0.262 for negative detections of plant DNA. This small difference also led to a significant overlap of the discriminant scores of the two groups (Figure 3), resulting in approx. 26.5% misclassified cases (i.e., false negatives and false positives taken together, also see above). The standardized canonical discriminant function coefficients (Table 3) indicate that “time post‐feeding” and “1,000 seed mass” had the strongest predictive power on the detectability of plant DNA. Those two factors as well as “protein content” were correlated with a negative detection. “Meal size” and “oil content,” in contrast, were correlated with a positive plant DNA detection.

**Table 2:** Classification of the DNA extracts via a discriminant analysis as “DNA detectable” or “DNA not detectable.” Positive—samples that tested positive for seed DNA. Negative—samples that tested negative. False positives/false negatives were samples that have been incorrectly assigned to the respective group

Seed species	Positive	Negative	False positive	False negative
*Capsella bursa‐pastoris* (61)	12	29	20	0
*Lolium perenne* (83)	5	62	11	5
*Rumex obtusifolius* (62)	13	30	17	2
*Senecio vulgaris* (66)	21	23	19	3
*Taraxacum officinale* (79)	21	35	19	4
*Trifolium repens* (60)	5	46	5	4
Total (411)	77	225	91	18

**Table 3:** Standardized canonical discriminant function coefficients to predict the detectability of seed DNA in regurgitates of carabid beetles. Note: as in this case a negative discriminant score predicts a successful detection of seed DNA, negative coefficients indicate an enhancement of detections and positive coefficients indicate a correlation with reduced detectability

Predictive variable	Coefficient
Digestion Time (hr)	0.982
1,000‐seed mass (g)	0.736
Oil Content (%)	−0.149
Protein Content (%)	0.322
Meal size (mg)	−0.397

**Figure 4:**
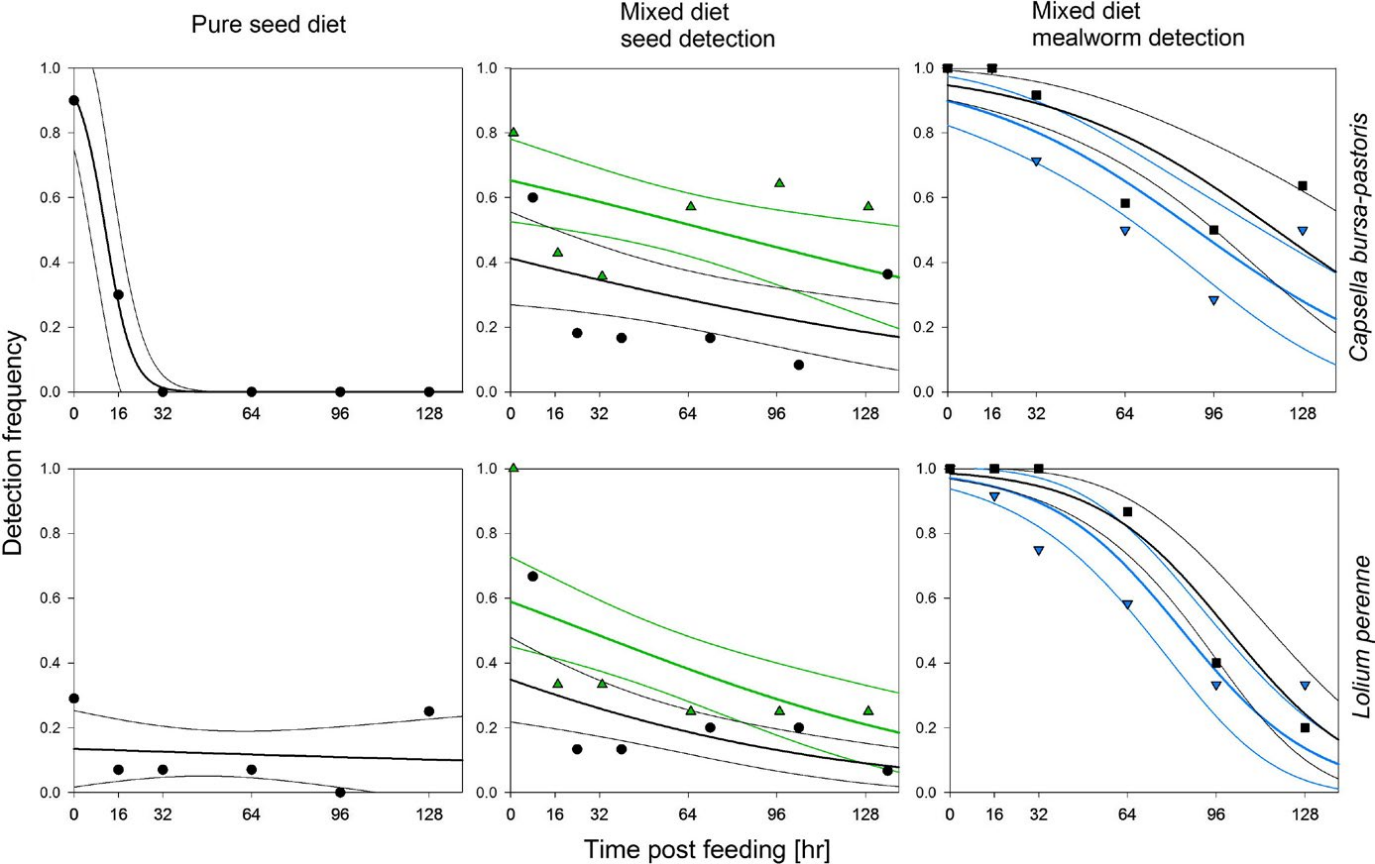
Detectability of seed and mealworm DNA in regurgitates of *Pseudoophonus rufipes* in a pure and mixed‐diet feeding scenario at different time points post‐feeding ranging from 0 to 128 hr after the consumption of their last meal (i.e., mealworm). Carabids were fed with seeds of either *Capsella bursa‐pastoris* (upper panel) or *Lolium perenne* (lower panel) first and then with mealworm—with a meal break of 1 and 8 hr (green for seeds, blue for mealworms), respectively. A minimum of 10 regurgitates per time point post‐feeding was tested with general plant primers and species‐specific ones for mealworm DNA. Observed detection rates are provided for the different time points for seeds as black circles (1 hr meal break) and green triangles (8 hr) and for mealworm as black squares (1 hr) and blue triangles (8 hr) along with fitted LOGIT models for the decrease in seed DNA detection success including the lower and upper 95% confidence intervals (thin lines)

**Figure 5:**
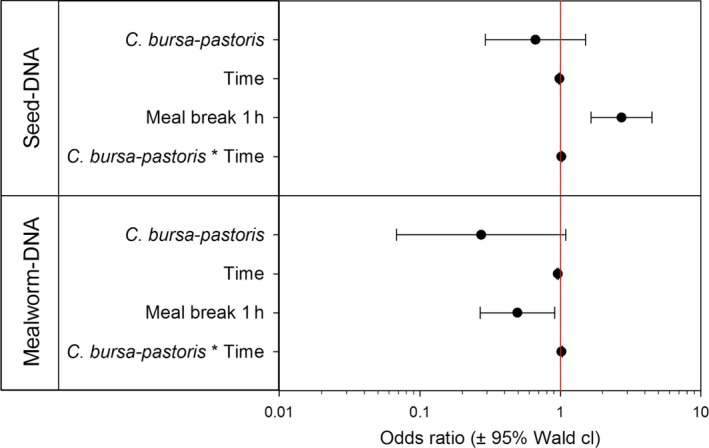
Odds ratios of food DNA detection compared to *Lolium perenne* and a meal break of 8 hr, respectively (OR = 1; vertical red line) inferred from the LOGIT regressions for seed and mealworm DNA detection

Due to the setup of the mixed‐diet experiment (meal break between seed and mealworm consumption), the digestion times for the DNA of consumed seeds are longer than for the mealworm for each time point post‐feeding (i.e., by 1 and 8 hr, respectively). For the sake of simplicity, we will refer to the digestion times in relation to the last meal (i.e., mealworm) in the text hereafter. However, the actual digestion time for the respective meal type (seed and mealworm, respectively) was included in the GLMs and for both models the same main effects (seed species, time, meal break) and interaction term (seed species*time) were considered in the final model based on significance of effects and informed by AICC. In the mixed‐diet experiment, both mealworm and seed DNA, of either *L. perenne* or *C. bursa‐pastoris,* could be detected for up to 128 hr post‐feeding. In general, detection rates of animal prey were higher than the ones of seeds (Figure 4). Detectability of plant DNA in the mixed‐diet experiment was considerably higher for a given time point post‐feeding than in the seed‐identity experiment, where carabids had consumed seeds only. In the mixed‐diet experiment, the detectability was 100% at 0 hr post‐feeding for both seed species (including a feeding interval of 1 hr between seed and mealworm) compared to 90 and only 30% for *C. bursa‐pastoris* and *L. perenne* in a pure seed diet. The GLM indicated a highly significant influence of the “length of the meal break” (1 hr vs. 8 hr; Wald χ^2^ = 15.328, *p* < 0.001) and time (Wald χ^2^ = 8.618, *p* = 0.003) on the detection probability of seed DNA. While the seed species, in this case *C. bursa‐pastoris* or *L. perenne,* had no significant influence on the outcome (Wald χ^2^ = 0.946, *p* = 0.331), the interaction between “seed species” and time was significant (Wald χ^2^ = 6.325, *p* = 0.012). Not only the detection rates of the seeds were influenced by the mixed diet, but also the probability of mealworm detection changed. For seed detection, the “length of the meal break” (Wald χ^2^ = 5.136, *p* = 0.023), time (Wald χ^2^ = 65.360, *p* < 0.001), and the interaction between “seed species” and time (Wald χ^2^ = 4.522, *p* = 0.033) had a significant influence on mealworm detection, but not the seed species per se (Wald χ^2^ = 3.368, *p* = 0.066). The odds ratios revealed an interesting effect of the meal break on the detection probability of the two food types. While the odds for seed DNA detectability were higher with the short, 1 hr meal break compared to the long one, the opposite was true for mealworm DNA (Figure 5). Comparisons of the estimated marginal means, however, showed only a significant difference in the detection probability depending on the “length of the meal break” for seed DNA (*p* < 0.001) but not for mealworm DNA (*p* = 0.054) or differences between the seed species (*p*_seed_ = 0.328, *p*_mealworm_ = 0.089).

## 4 DISCUSSION

The current study demonstrates that DNA of ingested seeds from different plant species can be detected in carabid regurgitates for up to 5 days post‐feeding under laboratory conditions. These findings indicate the potential of a sufficiently large time frame in which we can detect and identify food sources in field‐collected carabid beetles, particularly when consumption of a specific prey may date several hours or days prior to collection, as in case of a bad weather period for example. This is highly important when it comes to the analysis of field‐collected beetles, where the feeding frequency and exact time of actual consumption are usually unknown. The present results are in accordance with earlier experiments, showing extended detection periods/high detection rates of both seed (Wallinger et al., 2015) and animal prey in *P. rufipes* (Monzo et al., 2011; Waldner, Sint, Juen, & Traugott, 2013). These long prey DNA detection intervals may be ascribed to the omnivorous feeding mode of *P. rufipes* (Waldner et al., 2013). Like in most carabids, the digestive tract of *P. rufipes* is characterized by the capability to digest many different food sources, rather than being optimized for the effective breakdown of a certain prey type (Holland, 2002; Tooley & Brust, 2002). These carabids might therefore exhibit slower basal metabolic rates than comparably sized carnivorous arthropods. Moreover, the enzymatic breakdown of ingested plant tissue or seeds seems to be generally slower than that of insect tissue because plant material is harder to digest. Apart from prolonged DNA detection rates, plant food detectability does not generally seem to follow a logistic decline, as usually seen in the digestion of animal prey. In some cases, detection rates of plant food are already low at the beginning and we can see an increase in detection rates at late time points following an initial decrease, as for example in S. vulgaris. These findings are in accordance with earlier feeding experiments with herbivorous insects (Staudacher, Wallinger, Schallhart, & Traugott, 2011; Wallinger et al., 2015, 2013 ). The differences may be related to the nature of the digestion of plant tissue. Some plant‐specific metabolites could have an inhibitory effect resulting in low initial detection rates which can continue until detectability increases again during advanced digestion and associated lysis of plant cell walls or breakdown of plant secondary compounds. Altogether, this make the finding of appropriate models describing the digestion and DNA detectability of plant food challenging. This also prevented the possibility of fitting a single model describing the data of pure seed consumption together with that of the mixed diet in the present experiment. Based on experiences from former feeding experiments, where it had happened that DNA was still detectable at the end of the experiment and did not reach the base line, we already accounted for long detection rates (observation over five days post‐feeding) and were therefore able to characterize the digestion of most seeds in the pure diet very well. However, the additional consumption of mealworm increased detection rates dramatically. For example, in *C. bursa‐pastoris*, no seed DNA was detectable after 32 hr or later if only the seed was consumed, but with subsequent consumption of a mealworm, the detection rate remained at around 60% for five days, and again we saw an increase in detection rates following an initial decline. These aspects regarding DNA detection of plant food in arthropods, contrasting the knowledge of animal tissue digestion, clearly require further investigations.

As might be expected, seed DNA detectability had been negatively affected by time post‐feeding. However, the decrease in seed DNA detection rates in beetles regurgitates turned out to be seed species dependent, thus confirming earlier presumptions that seed identity might play a significant role (Wallinger et al., 2015). The same was true for different plant species in root feeding insects (Wallinger et al., 2013). While the detectability of some species dropped within the first 32 hr, the DNA of others was still detectable at the maximum period tested (i.e., 5 days post‐feeding). Accordingly, there were significant differences when pairwise comparing overall DNA detection rates in the single food‐source experiments. Seed mass and protein content turned out to have a negative effect on the detectability of plant DNA, whereas higher oil content seemed to result in longer detection periods. The latter was in accordance to the findings of Thomas, Jarman, Haman, Trites, and Deagle (2014), who reported enhanced prey detection for lipid‐rich prey as well. This indicates that high lipid content of prey is associated with diminished tissue breakdown and accordingly slower DNA degradation during digestion. That means, in turn, that the ratio of lipid‐rich seeds is likely to be overestimated compared to other ones, an issue that needs to be considered when it comes to the analysis of field‐collected data. However, unlike here where the protein content of seeds was negatively correlated with DNA detectability, in the study of diet estimates of Thomas et al. (2014), also protein content was correlated with higher detection rates, which the authors ascribed to the fact that increased body protein is associated with elevated levels of mitochondrial DNA, which had been targeted in their work. This factor does not play a role here, as the study by Thomas et al. (2014) was based on animal tissue, whereas the present study used the *trn*L exon, part of the chloroplast DNA, to detect DNA from plant tissue. The “meal size” in earlier feeding experiments with *P. rufipes* referred to the amount of consumed seeds of two plant species with rather similar size, which did not affect plant DNA detection (Wallinger et al., 2015). However, when comparing the effect of “meal size” between different seed species, one should also consider the high variation in size and biomass between seeds. Therefore, in the present experiments, “meal size” refers to the number of consumed seeds * average seed mass. Here, it had a positive effect, indicating the importance of the biomass of a certain prey type consumed by a carabid beetle. We want to point out, that these findings on the influence of seed traits here are based on data from six seed species only, covering just a fraction of the variability of those plant traits. Unfortunately, we have a coincidental correlation of “1,000 seed mass” and “oil content” in the present subset due to three species showing a low (<7%) and the three other ones a high (>26%) oil content, respectively, which would be not the case if a wider range of plant species is considered. Thus, a slight reduction in the predictive power of the discriminant analysis due to multicollinearity could not be excluded and we ran the discriminant analysis also with either one variable excluded (data not shown). As expected, the coefficient of the respective other factor increased in these cases, but overall a decrease in the percentage of correct classifications was observed—especially in the already difficult prediction of negative samples. Thus, we do not think that the results of the presented discriminant analysis are strongly biased by this fact and decided to keep both explanatory variables. Still, it would be highly interesting to extend the knowledge on the influence of different seed traits to better understand digestion of seeds by carabids and thus to improve in the long run the models predicting the digestion and detectability of plant DNA over time.

In general, the recovery of food DNA has been shown to be affected by numerous variables (King, Read, Traugott, & Symondson, 2008) and the observed differences might be ascribed to biological reasons or introduced via the methodological approach (Thomas et al., 2014). Potential methodological biases are, for example, different efficiencies due to varying primer binding sites (Sipos et al., 2007) or differences in the length of the amplified fragment (Sint et al., 2011; Waldner et al., 2013). They can be minimized by a prudent study design including an appropriate assay selection. For example, in the present case, binding sites as well as expected fragment length had been the same for all seed species. On the other hand, biases due to biological factors influencing food DNA detectability are rooted in variation inherent to the system. Primarily, they can be ascribed either to differences in target DNA density in the consumed tissue (Deagle & Tollit, 2007) or in prey DNA survival during digestion. Especially prey DNA survival depends on many factors such as temperature (Hosseini, Schmidt, & Keller, 2008), additional prey consumed (Penry & Juman, 1987), or prey identity, as described above. In case of the latter, the use of correction factors, evaluated by measuring species‐specific DNA recovery rates, is suggested to improve the estimates (Greenstone et al., 2014). However, developing such correction factors is not always that straightforward. In the present case, it would require multiple extensive, time‐consuming feeding trials including every single species that carabids potentially might prey on, or at least an extensive, representative selection to potentially identify certain seed traits as robust predictors. And even if we can generate such correction factors, still not all biases are feasible to mitigate for every potential food source in combination with the multiple factors such as meal size, mixed feeding, and many more. When it comes to the molecular identification of specific taxa to unravel trophic interactions in the field, in some cases a diagnostic approach via species‐specific primers may thus represent an advisable alternative to NGS and the use of general primers, provided that the primers are balanced in their sensitivity and the potential food sources are restricted to a certain number of known seed species. However, even when many consumed species of an unknown prey spectrum make DNA metabarcoding and accordingly the use of general primer necessary, the identification of seed DNA can clearly provide informative data, as for example correctly identifying frequently consumed seed species. This applied all the more since the differences in DNA detection observed in the seed‐only experiment were no longer evident in the mixed‐feeding setup, indicating that the seed type will likely play a minor role in field‐caught carabids. Moreover, food DNA can still be detected when present at low levels so that the unequal digestion of DNA from different prey species will not necessarily translate into significant biases in a semiquantitative approach based on presence/absence data (Deagle & Tollit, 2007). Therefore, the identification of food DNA in regurgitates seems appropriate for determining the carabid diet composition in the field. In this context, we recommend high numbers of replicates.

The subsequent feeding of a mealworm after seed consumption led to an increased detectability of seed DNA in the regurgitates and an extension of the detection time post‐feeding compared to a diet exclusively on seeds: initial detections rates of *C. bursa‐pastoris* and *L. perenne* at 0 hr post‐feeding were higher and the decrease in detectability was attenuated, especially when the seed and insect food were consumed immediately (1 hr) one after the other. This effect was less pronounced at an extended meal break of 8 hr between seed and mealworm consumption. According to the optimal digestion theory, a meal is expected to disappear faster with access to food following it, than after the target meal only, with no subsequent opportunity to feed (Penry & Juman, 1987). This effect was confirmed in an experiment conducted by Weber and Lundgren (2009) who were able to detect DNA of the Colorado potato beetle over a longer time span if the predator was not allowed to feed on other prey thereafter. One might thus expect shorter retention times in the guts, resulting from physical effects of the first meal being displaced by subsequent food. Earlier studies on the influence of dietary mixing of different animal prey types, however, have shown varying influences on the detectability of the target prey, ranging from positive (Fournier et al., 2006; Harper et al., 2005), to none (Lövei, Sopp, & Sunderland, 1987, 1990 ), to negative (Weber & Lundgren, 2009). In the current experiment, we found a clear positive effect of subsequent mealworm consumption on the post‐feeding detection of seed DNA. At the same time, the presence of seed DNA did not seem to have strong effects on the detection of the animal prey when comparing them with other feeding experiments with *P. rufipes* (Waldner et al., 2013) or employing the same mealworm primers as we did (Sint et al., 2011), although the detectability of the mealworm DNA in the present experiment was slightly higher when the meal break between seed and animal prey was longer. Altogether, this indicates that plant and animal food can be detected over a considerable period of time post‐consumption in omnivorous carabids. That is highly relevant also in field‐caught beetles, where it remains unclear whether they consumed only one prey type or several ones and how long ago the feeding has happened. This potentially opens the possibility to investigate the full dietary spectrum of omnivorous carabid beetles under field conditions with molecular methods, delivering important insight into their feeding behavior and consequently increase the knowledge on their provision of ecosystem services.

It should be taken into account that our conclusions are based on the analysis of a single carabid species so far, tested under laboratory conditions. Still, in choosing, *P. rufipes* we have selected a species that is not only dominant in ground beetle communities but also a representative, in being omnivorous and a chewing feeder like most carabids in arable land. The detection times observed can serve as guidelines for interpreting field‐derived data, although several factors that are present in the field may additionally influence the detectability of food DNA, such as fluctuating temperatures. High ambient temperatures have been found to effectively decrease detection rates of prey DNA (Hoogendoorn & Heimpel, 2001; Hosseini et al., 2008; von Berg, Traugott, & Scheu, 2012). Carabids, however, are surface active and many of them are nocturnal (Thiele, 1977), which implies that they are hiding during the day and might therefore not be subjected to strong daily temperature fluctuations. Thus, the effect of temperature on DNA detection rates probably might be less important for interpreting molecular trophic data. Still, there are various other aspects that need to be taken under consideration, such as the influence of activity levels, the ontogenetic status on digestion or sex‐specific differences as reported by Lundgren (2009), which might be specifically examined in the field to account for the variability present in natural settings. Future work, testing the detection intervals in mesocosms or a field experiment would represent the next logical step toward a better interpretation of field data based on carabid regurgitates.

In vertebrate studies, non‐invasive approaches to obtain biological material for molecular analysis have long been preferred (Waits & Paetkau, 2005); however, these approaches have only recently been adopted for use in invertebrate studies (Lefort, Boyer, Worner, & Armstrong, 2011; Raso et al., 2014; Seeber, Rief, Seeber, Meyer, & Traugott, 2010; Sint, Thurner, Kaufmann, & Traugott, 2015). Apart from ethical reasons, using carabid regurgitates instead of whole‐body DNA extracts has numerous advantages. First of all, the avoidance of killing the beetles is essential for example in surveys of small populations or when working with beneficial, rare, or endangered species. In addition, the application of this nondestructive approach offers the opportunity to monitor dietary changes in carabid beetles, which can be dependent upon different seasons (Holland, 2002; Hulme, 2002) or vary ontogenetically (Lundgren et al., 2009; Thiele, 1977). In comparisons of fecal, gut content, and regurgitate samples, prey DNA detection rates were the highest for regurgitates (Frei, 2018; Kamenova, Mayer, Coissac, Plantegenest, & Traugott, 2017; Waldner & Traugott, 2012). This indicates that they constitute a good source of prey DNA. Also in terms of fixation and storage, regurgitates are an advantageous sample type, as they can be directly dissolved in lysis buffer or quickly preserved in ethanol, which immediately inhibits the activity of digestive enzymes. Preserving invertebrates as a whole, in contrast, has shown to be less efficient in respect to the quality of prey DNA (Waldner & Traugott, 2012). This might be explained by the longer time ethanol needs to penetrate and fixate the gut content. Preserving regurgitates in ethanol will be especially useful for projects conducted in remote areas (e.g., alpine environments, tropics) where freeze‐storage of samples is difficult. Finally, regurgitates contain comparable low concentrations of predator DNA. This is especially beneficial when DNA extraction methods are used where the overall amount of nucleic acids that can be isolated is limited (e.g., all silica‐based methods). Furthermore, large amounts of nontarget DNA and inhibitors present in whole‐body insect extracts can be a particular problem in molecular diet studies, where predator DNA is often present in great excess of food‐derived DNA. The predominance of one DNA template within a single sample can bias or restrict molecular analysis (Polz & Cavanaugh, 1998), especially if general primers are employed which might co‐amplify the consumer DNA (Krehenwinkel, Kennedy, Pekár, & Gillespie, 2016). Aside from carabid beetles, regurgitation is also common in other arthropod taxa, such as spiders (Kaestner, 1993), grasshoppers (Sword, 2000), or soil‐dwelling invertebrate predators (Juen & Traugott, 2007), indicating a wide applicability of the current approach.

In conclusion, the present data show that with this noninvasive approach, carabid consumption of various seed species can be molecularly detected for at least five days post‐feeding under laboratory conditions. Although detection times may vary under field conditions, this novel approach provides an opportunity to detect seed‐feeding and integrate this knowledge into food web ecology for carabid beetles. Our findings indicate that plant identity, chemical composition, and meal size can affect DNA detection of consumed seeds. The results from the mixed‐feeding experiment suggest that the omnivorous feeding mode of carabids may lead to prolonged detection intervals of consumed seeds, thus enlarging the potential time window for successful food detection compared to what has been previously assumed. The species‐specific differences observed seem to play a minor role in a mixed diet, which is natural for many carabid species. On the basis of broad tolerance margins and a high sample number, good estimates of trophic interactions between carabids and their seed prey can be drawn. Altogether, this novel approach represents a promising opportunity to identify seed‐feeding species and integrate this knowledge into food web ecology for carabid beetles. It will enable us to investigate the overall potential of carabid beetles as weed biocontrol agents in arable land. Apart from identifying carabid key species, it will be possible to evaluate the success of facilitation measurements in respect to a robust and resilient provision of this ecosystem service, therefore significantly contributing to international goals in reducing pesticide applications without compromising crop yield.

## CONFLICT OF INTERESTS

None declared.

## AUTHORS CONTRIBUTION

M.T. and C.W. conceived and designed the study. R.M. and C.W. performed the feeding experiments. R.M was responsible for DNA extractions and PCR screening for the single species experiment. Y.G. performed laboratory work regarding the mixed‐feeding experiment. D.S analyzed the data and contributed to the first draft. C.W. wrote the manuscript, which all authors revised and finally approved to be published.

## DATA AVAILABILITY

The data of the feeding experiments and molecular analysis have been uploaded to DRYAD Provisional http://https:/doi.org/10.5061/dryad.1n4f0b3.
