# Supplementary material for: The effect of plant identity and mixed feeding on the detection of seed DNA in regurgitates of carabid beetles
Source: Ecol Evol. 2018 Oct 25;8(22):10834–46. doi: 10.1002/ece3.4536 (PMC6262922; doi:10.1002/ece3.4536)
Supplement: Supplementary file 1 [file ECE3-8-10834-s001.docx]

**Table S1** Numbers of regurgitates going in to the analysis for the species identity experiment at the different time points post-feeding

|  |  |  |  |  |  |  |  |
| --- | --- | --- | --- | --- | --- | --- | --- |
| **Seed Species** | **Regurgitates per Timepoint post-feeding [h]** | | | | | | **Total** |
|  | **0** | **16** | **32** | **64** | **96** | **128** | **numbers** |
| *Capsella bursa-pastoris* | 10 | 10 | 10 | 10 | 10 | 11 | 61 |
| *Lolium perenne* | 14 | 15 | 14 | 14 | 14 | 12 | 83 |
| *Rumex obtusifoius* | 10 | 10 | 10 | 10 | 10 | 12 | 62 |
| *Senecio vulgaris* | 10 | 10 | 10 | 10 | 15 | 11 | 66 |
| *Taraxacum officinale* | 12 | 15 | 13 | 13 | 14 | 12 | 79 |
| *Trifolium repens* | 10 | 10 | 10 | 10 | 10 | 10 | 60 |
| **Total Numbers** | 66 | 70 | 67 | 67 | 73 | 68 | 411 |
