# Supplementary material for: The effect of plant identity and mixed feeding on the detection of seed DNA in regurgitates of carabid beetles
Source: Ecol Evol. 2018 Oct 25;8(22):10834–46. doi: 10.1002/ece3.4536 (PMC6262922; doi:10.1002/ece3.4536)
Supplement: Supplementary file 2 [file ECE3-8-10834-s002.docx]

**Table S2** Numbers of regurgitates going in to the analysis for the mixed feeding experiment at the different time points post-feeding

|  |  |  |  |  |  |  |  |  |
| --- | --- | --- | --- | --- | --- | --- | --- | --- |
| **Seed Species** | **Feeding break [h]** | **Regurgitates per Timepoint post-feeding [h]** | | | | | | **Total** |
|  |  | **0** | **16** | **32** | **64** | **96** | **128** | **Numbers** |
| *Capsella bursa-pastoris* | 1 | 10 | 14 | 14 | 14 | 14 | 14 | 80 |
| *Lolium perenne* | 1 | 9 | 12 | 12 | 12 | 12 | 12 | 69 |
| *Capsella bursa-pastoris* | 8 | 10 | 11 | 12 | 12 | 12 | 11 | 68 |
| *Lolium perenne* | 8 | 15 | 15 | 15 | 15 | 15 | 15 | 90 |
| Total Numbers |  | 44 | 52 | 53 | 53 | 53 | 52 | 307 |
